# Eco-Friendly approach for enhancing functionality of PET/C blended fabric with ZnO NPs

**DOI:** 10.1038/s41598-025-15063-z

**Published:** 2025-08-24

**Authors:** Naser Gad Al-Balakocy, Eman F. Ahmed, Sara H. Mansour

**Affiliations:** 1 Protein & Manmade Fibers Department, National Research Centre, Dokki, Cairo Egypt; 2 Chemistry of Natural and Microbial products Department, National Research Centre, Dokki, Cairo Egypt; 3 National Research Centre, Dokki, 12622 Cairo Egypt

**Keywords:** PET/C blended fabric, Pectinase, Enzymatic hydrolysis, ZnO NPs, EDX, SEM, FT-IR, Antimicrobial, UPF., Biotechnology, Chemical biology, Microbiology, Materials science

## Abstract

The current study explores at the viability of employing enzymatic treatments to activate fabric surfaces and enable for the long-term loading of zinc oxide nanoparticles (ZnO NPs) onto PET/C blended fabrics. The fabric was treated with pectinase degrading enzyme, manufactured by a locally identified fungus from agriculture waste.; the active strain was genetically identified as *Aspergillus foetidus* (NR_163668.1). Factors affecting the activation of textile material with pectinase enzyme (incubation time and concentration) were studied. Parent and pretreated textiles loaded with ZnO NPs were investigated using Scanning Electron Microscopy (SEM), Energy Dispersive X-Ray (EDX), and Fourier Transformed Infrared Spectroscopy (FT-IR). Antibacterial activity against *Staphylococcus aureus*, *Escherichia coli*, *Bacillus subtilis*, *Pseudomonas aeruginosa* and *Candida albicans* by using inhibition zone and shake flask methods was evaluated. UV protection efficacy of activated and ZnO NPs loaded textiles were assessed. The impact of different concentrations of the enzyme and different periods of pectinase incubation with the textile revealed that the highest activity of the partially purified enzyme (41.4 U/ml) was got at the first 15 min and an increase in the activity of pectin degrading enzyme from 20.5 U/ml by 0.8 g/l enzyme to 70.6 U/ml by the use of 4 g/l pectinase. *Candida albicans* and *Pseudomonas aeruginosa* were got 0.84 × 10^8^ and 0.6 × 10^8^ CFU/ml using shake flask method respectively. The pretreated and ZnO NPs loaded fabrics demonstrated exceptional and durable antibacterial activity using inhibition zone and UV protection efficiency, even after five washing cycles.

## Introduction

 Polyester/cotton blended (PET/C) fabrics are commonly used in a variety of applications, including garments, furniture, and industrial textiles. While polyester is known for its durability and wrinkle resistance, it is also susceptible to pilling and creasing, which can have an impact on the fabric’s overall appearance and function. Cotton, on the other hand, is airy and soft, although it is susceptible to shrinking and wrinkles. As a result, researchers have been investigating the production of modified polyester materials with improved functional and comfort features^1–4^.Textile finishing is a crucial step in the textile manufacturing process, as it enhances the performance, appearance, and functionality of fabrics. In recent years, nanoparticles (NPs) have gained significant attention for their potential applications in textile finishing. NPs offer unique properties, such as improved antimicrobial activity, UV protection, and self cleaning capabilities, which can be exploited to create novel textile finishes^5–8^.

One interesting option is to incorporate Zinc Oxide (ZnO) nanoparticles into polyester/cotton hybrid fabrics. ZnO is a well-known antibacterial agent that has a wide range of applications, including wound dressings, medical implants, and food packaging. ZnO nanoparticles’ distinctive features, such as high surface area, reactive surface chemistry, and propensity to create reactive oxygen species (ROS), make them a promising alternative for improving the antibacterial and UV-protective qualities of polyester fabric^9–15^. Challenges: Because of the intricacy of the synthesis process, producing PET/C blended fabric finished with ZnO NPs on a large scale remains difficult. Integration of ZnO NPs into commercial textile products is difficult due to their comparatively high production costs. Stability: To guarantee ZnO NPs’ long-term efficacy, their stability on the fabric surface must be increased^16–18^.

Carboxylic acid (COOH) and hydroxyl (OH) groups are potential sites for binding of NPs, the formation of OH and COOH groups on polyester fabric surface is a chemical reaction that can be induced by various mechanisms, including hydrolysis, or plasma treatment^19–22^. The resulting functional groups can significantly impact the properties of the fabric, making it suitable for various applications in medical textiles, coatings, and biodegradable materials. However, the implementation of these techniques is limited due to the complexity of the technologies involved.

Several methods for surface modification of PET/C blended fabrics have been published in the literature, such as alkali hydrolysis^23,24^, aminolysis^25^, graft co-polymerization^26^, quaternary ammonium salts^12^, and others. These were designed to chemically alter the surface of polyester. Alkali treatment results in considerable weight loss (13–15%) and loss of strength. Furthermore, alkali processing produces a large amount of waste, which raises the cost of textile effluent treatment. High weight add-ons (up to 50 wt%) might impact the look and air permeability of fabrics after application. Several novel ways of changing the surface of PET textiles have been described in the literature, including plasma^27^, UV irradiation^28^, excimer, and their combination^29^. Despite all of these strategies, the permanence of such surface treatments remains unclear. Furthermore, these developing approaches are still in their early stages and will require additional development before they can be commercialized.

To address the binding efficiency issue, researchers have been exploring the development of enzyme activated PET/C blended fabrics. Enzymes are biological molecules that can catalyze specific chemical reactions, allowing them to break down or modify the fabric’s molecular structure. In this context, PET/C blended fabrics can be activated using enzymes, improving their antibacterial and durable press qualities^30^.

Enzyme activated PET/C blended textiles have the following advantages^31,32^: Better durable press: Even after several washings and dryings, the cloth keeps its appearance and form. Improved antibacterial qualities: The cloth inhibits the growth of fungi, bacteria, and other germs. Better breathability and moisture transmission are made possible by the fabric. Decreased pilling: The cloth looks better overall and lessens the production of pills. Sustainability: Compared to conventional chemical treatments, the enzyme assisted finishing method is more ecologically friendly.

Conventionally the scouring process of PET containing cotton carried out by treating the fabric with caustic soda and sodium hydroxide. The use of traditional strongly alkaline process can have a detrimental effect on fabric weight and on the environment. Enzymatic scouring makes it possible to effectively scour fabric without negatively affecting the fabric or the environment. Hydrolysis by enzymes such as pectinase promotes efficient interruption of the matrix to achieve good water absorbance without the negative side effect of cellulose destruction. This process is called bio-scouring. It breaks down the pectin in the cotton and thus assists in the removal of waxes, oils and other impurities^33–37^. The fabric gives better wetting and penetration properties, making subsequent bleach process easy and resultantly giving much better dye uptake.

The aim of this study is to investigate the use of enzyme assisted finishing by ZnO NPs to enhance the durable functional antimicrobial and UV protective properties of PET/C blended fabrics. This article will use pectinase enzyme, to modify the fabric’s molecular structure. Also, will study the characterization of the modified polyester fabric using a range of techniques, including SEM, EDX, XRD, and FT-IR to show the effect of pretreatment of PET/C blended fabrics with pectinase before loading with ZnO NPs on the fabric’s properties. The results of this study will provide valuable insights into the potential applications of enzyme-activated polyester/cotton blended fabrics in various industries, including textiles, clothing, and upholstery by using facile and Eco-friendly approach.

## Experimental work

### Materials

#### Fabrics

Polyester/Cotton Blend (PET/C) 50/50 fabrics used throughout this study were in the form of filament woven fabric cloth made from filament yarns. They were kindly supplied by Misr polyester Co., Kafr EL-Dwar, Egypt. The fabrics were scoured at 80 °C for 45 min and M: L, 1: 5. with solution containing 2 g/L nonionic detergent, washed with cooled water, squeezed, and finally air dried for 2 h.

#### Nanoparticles

ZnO dispersion was commercial product (particle size = 20 nm) was purchased from Sigma – Aldrich.

#### Enzyme

Acid pectinase enzyme used through this work was produced from a fungus which was isolated locally from a garden soil. The active strain was genetically identified as *Aspergillus foetidus* (NR_163668.1).

#### Microorganisms

The pathogenic microorganisms used in this study were *Staphylococcus aureus*,* Bacillus subtilis*, *Pseudomonas aeruginosa*, *Escherichia coli* and pathogenic yeast *Candida albicans*. The organisms were prepared from fresh overnight nutrient agar medium that were incubated at 37 °C for 24 h.

#### Culture medium

Modified nutrient agar medium is composed of the following ingredients (g/L): peptone (10.0), beef extract (5.0), NaCl (5.0), and agar (20.0). The pH was adjusted to 6.8. This medium was sterilized for 20 min at 121 °C under pressure. Potato dextrose broth (PDB) was used as a submerged fermentation medium for production of pectinase enzyme.

### Methods

#### Production of pectinase

The enzyme was produced in a maximum yield, 138 U/mg at pH 4.5 after 5 days of incubation at 37 °C in a submerged fermentation medium which contained wheat bran, (4%), yeast extract (1%) and EDTA (0.2%).

#### Application of the partially purified enzyme on textile

##### Optimization of incubation time and the concentration

The enzyme was incubated at 50 °C with a piece of the textile (0.75 g) at a range of time (15–60 min). Finally, the activity of the enzyme was evaluated. Further, different concentrations of the enzyme (0.8–4.8 g/l) were incubated with (0.3 g) textile at 50 °C for 15 min and the activity of the enzyme was calculated.

##### Preparation of activated polyester fabrics

**Fig. 1:**
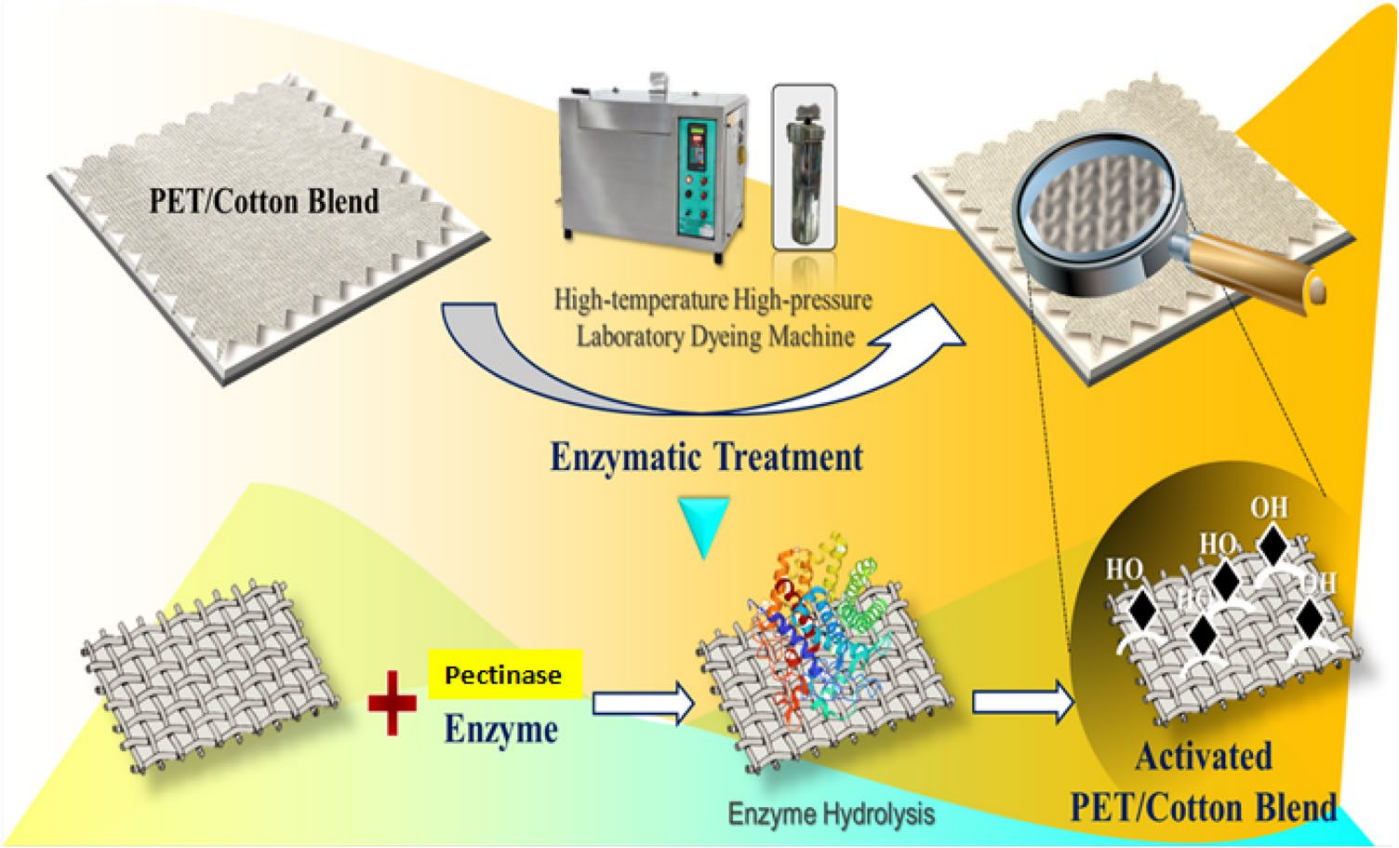
Experimental representation for enzymatic surface activation of PET/C blended fabric with pectinase enzyme.

The treatment of PET/C blended fabrics with pectinase was carried out using a high temperature high pressure laboratory dyeing machine (model LL - India). The required amounts of pectinase were placed in stainless steel bowls (5.0 g/l in aqueous solution), the fabrics samples were immersed in the aqueous solution its initial pH = 7.5 adjusted to 4.0 with Acetic Acid), and the sealed bowls were rotated in a closed bath containing ethylene glycol at 50 °C. The material: liquor ratio (M: L, 1:15). The bath temperature increased from room temperature 25 °C at rate of 5 °C/min. After 40 min, the enzymatic treatment was then terminated by raising the pH to 10 by using Na_2_CO_3_; the samples were removed from the bath, rinsed repeatedly with distilled hot and cold water, and then the treated fabric samples allowed to dry in the open air (Fig. 1).

The extent of biodegradation was estimated from the weight loss (WL) of 3 fabric samples based on the following equation:

1 $$WL{\text{ }}\left( \% \right){\text{ }}={\text{ }}\left[ {{W_1} - {\text{ }}{W_2}/{\text{ }}{W_1}} \right]{\text{ }} \times {\text{ }}100$$

Where: W_1_ and W_2_ are the weights of the samples before and after enzymatic treatments.

#### Preparation of PET/C fabrics loaded by ZnO NPs

**Fig. 2:**
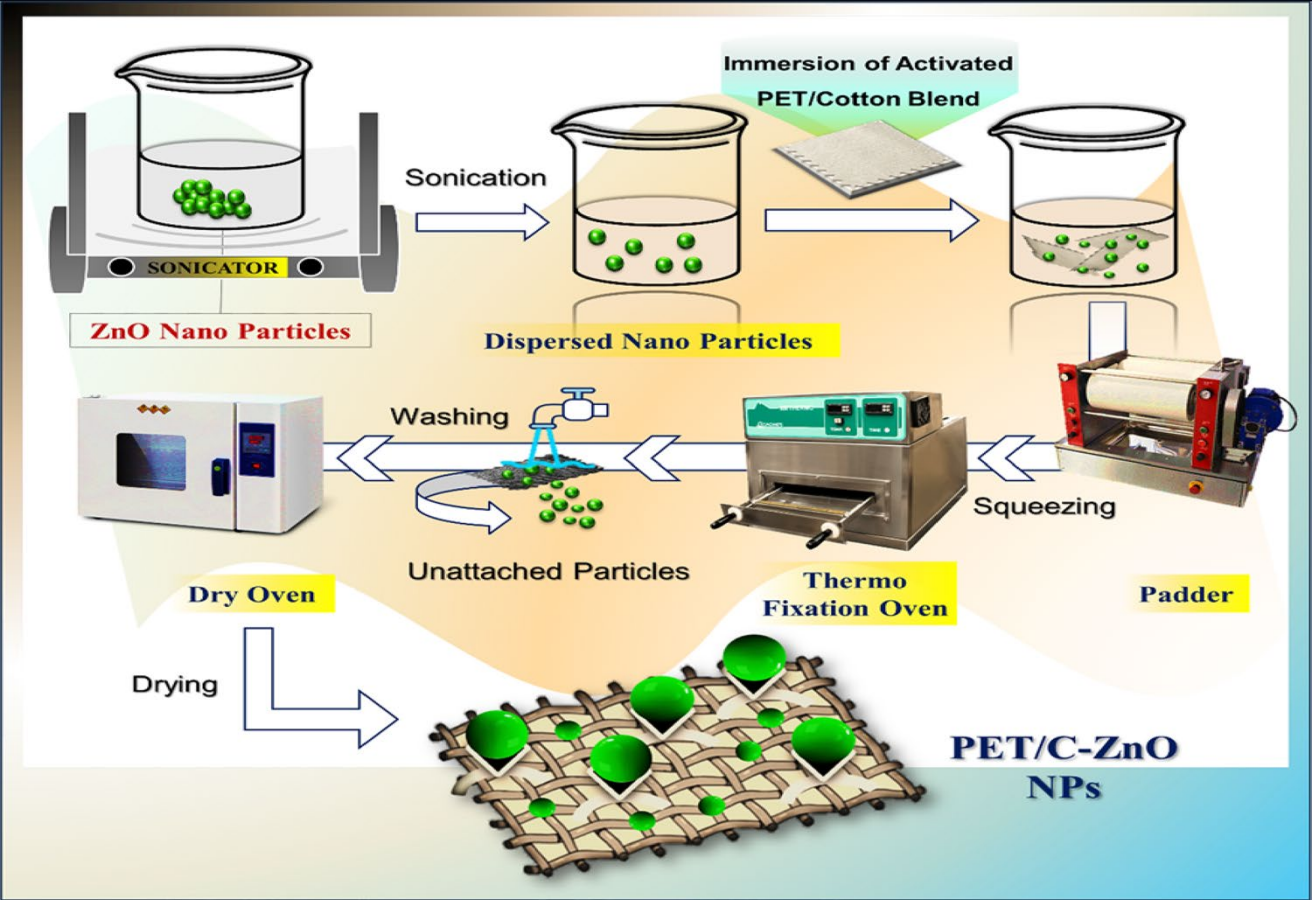
Experimental representation for surface decoration of activated PET/C blended fabric by pectinase and loaded with ZnO NPs.

The activated PET/C Blended fabrics by pectinase and hydrolyzed fabrics before enzymatic treatment were immersed in the ZnO NPs 0.5% dispersion, the samples were padded for 5 min then squeezed to a pickup of 60% (wt/wt) of the solution, and dried in air at 22 °C (laboratory temperature) for 24 h, and finally cured in an oven at 150 °C for 15 min (Fig. 2).

### Analysis

#### Inhibition zone method

Different pathogenic strains (Gram-positive *Staphylococcus aureus*, *Pseudomonas aeruginosa*, *Bacillus subtilis*, Gram-negative, *E. coli* and non-filamentous fungus *C. albicans*) were cultured in nutrient agar medium in Petri dishes. Specific weight of the textile (0.5 g) was added on the medium which was incubated for 24 h at 37 °C. Then, the inhibition zone was noticed and measured.

#### Evaluation of cfu/ml of pathogens

Different pathogenic strains (*Pseudomonas aeruginosa* and *Candida albicans*) were used to inoculate 50 ml nutrient broth medium which was prepared in 250 ml shake flasks. Specific weight of the textile (0.5 g) was added and the media were incubated for 24 h at 37 °C. After that, an inoculum (10µL) from a diluted culture filtrate was used to inoculate nutrient agar medium in Petri dishes and incubated at 37 °C for 24 h. The cells were counted and finally, the (CFU/ml) was calculated according to the following equation:

2 $$\begin{aligned} CFU/ml= & \frac{Number{\text{ }}of{\text{ }}colonies \times {\text{ }}dilution{\text{ }}factor}{Volume{\text{ }}of{\text{ }}plated{\text{ }}culture} \\ & & {\kern 1pt} & & \\ \\ \end{aligned}$$

#### Washing durability

To investigate the washing durability of antibacterial activity and UV protective properties, the antibacterial rate and UV protection factor (UPF) of NPs functionalized PET/C blended fabrics were evaluated after 1 and 5 washing cycles, as per the AATCC test method (61-1989). In a typical washing cycle, one wash is equal to five hand washes. Samples were washed using 2.0 g/l detergent at 70 °C for 40 min and M: L, 1: 50.

#### Carboxylic content

The carboxylic content of activated PET/C blended fabrics by pectinase enzyme was measured according to the method described in^38^.

#### Surface structure

The morphology of all fabric samples were characterized by a JEOL-Model JSM T20 scanning electron microscope (SEM), operating at 19 kV to obtain photomicrographs of fabrics surface. Gold layer was deposited on the samples before the analysis.

#### FT-IR

The chemical structure was determined using the Fourier transformation infrared (FT-IR) spectrometer, model NEXUS 670, NICOLET USA. The measurements were carried out in spectral range from 4000 to 500 cm^−1^. Reflection percentage measurement technique was applied (R %) to all investigated samples.

#### UPF factor

UPF factor was measured using UV- Shimadzu 3101 PC-Spectrophotometer. It is a double beam direct ratio measuring system. It consists of the photometer unit and a pc computer. UPF factor was determined according to the method described in Australian/New Zealand standard AS/NZS 4399: 1996^34^.

UV protection and classification according to AS/NZS 4395:1996.

**Table:** 

UVP	UPF classification
Excellent	40, 45, 50, 50+
Very good	25, 30, 35
Good	15, 20
Non- ratable	5, 10

## Results and discussion

### Application of pectinase enzyme in textile industry

For fabrics made from cotton or blends, the warp threads are coated with an adhesive substance known as “size”, to prevent the threads breaking during weaving. Although many different compounds have been used to size fabrics, starch and its derivatives have been the most common sizing agent^39^. After weaving, the size must be removed again in order to prepare the fabric for dyeing and finishing. This process (desizing) must be carried out by treating the fabric with chemicals such as acids, alkali or oxidizing agents.

The chemical treatment was not totally effective in removing the starch (which leads to imperfections in dyeing) and also results in a degradation of the cotton fiber resulting in destruction of the natural soft feel, or hand, of the cotton. However starch breaking enzymes are preferred for desizing due to their high efficiency and specific action. Using amylase enzymes for the removal of starch is one of the oldest enzyme applications^40^. bio-scouring, it breaks down the pectin in the cotton and thus assists in the removal of waxes, oils and other impurities. The fabric gives better wetting and penetration properties, making subsequent bleach process easy and resultantly giving much better dye uptake.

### Molecular identification of fungal strains

**Fig. 3:**
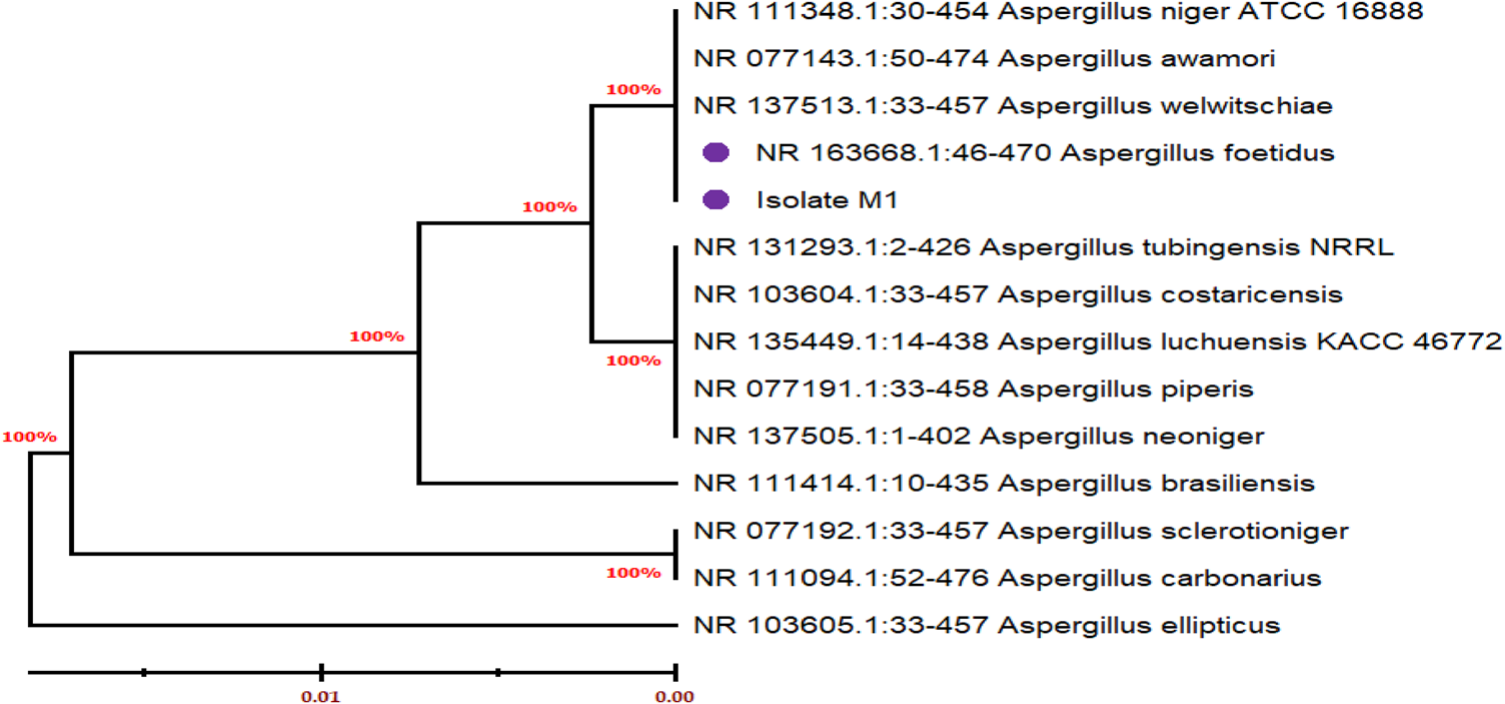
Phylogenetic tree based on partial ITS sequences, showing the relationship between isolate No (M1) *Aspergillus foetidus* and other species belong to the genus Aspergillus. The tree was constructed using the MEGA11 and neighbor-joining method.

The results in Fig. 3 confirmed that the active pectinase producer fungus was closely related to *Aspergillus foetidus* strain with the Accession number (NR_163668.1).

### Effect of incubation time of the precipitated enzyme on degrading pectin

**Fig. 4:**
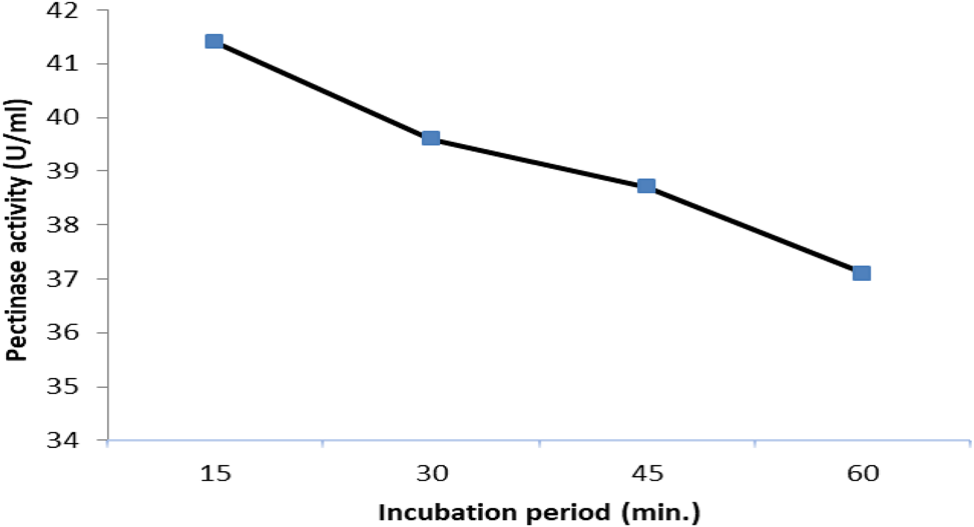
Impact of incubation time of the precipitated enzyme on degradation of fabric pectin.

The results in Fig. 4 confirmed that the highest activity of the precipitated enzyme (41.4 U/ml) was got at the first 15 min. After that a slight decrease in activity was obtained this result is referred to the denaturation of the enzyme that occurred by time which led to a reduction in the activity. From this result we could conclude that the optimum time of incubation of pectin degrade from fabric to get the best treatment is at the first 15 min.

### The effect of pectinase concentrations on degrading pectin

**Fig. 5:**
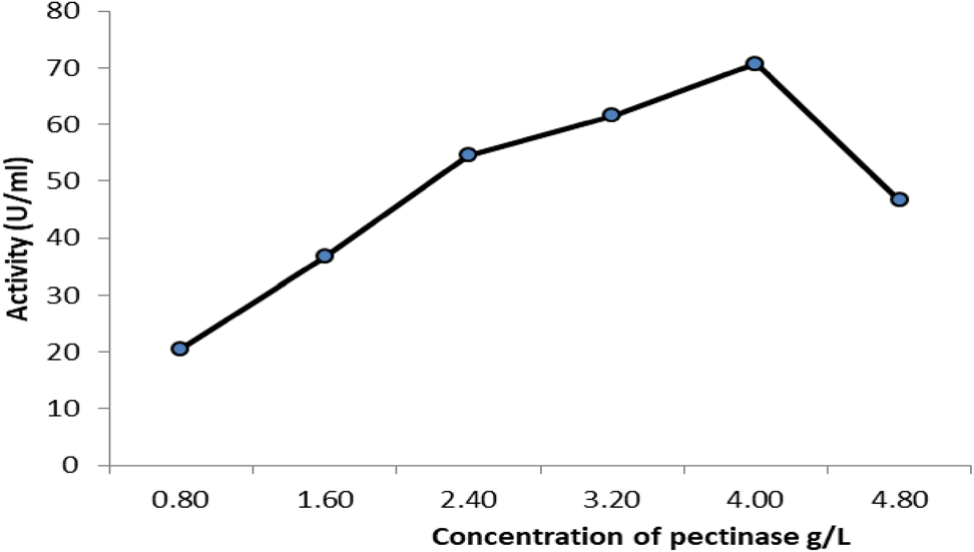
Effect of ascending levels of pectinase on degradation of pectin loaded on PET/C blended fabric.

 The data illustrated in Fig. 5 showed a clear increase of the activity of the pectin degrading enzyme from 20.5 U/ml by 0.8 g/l enzyme to 70.6 U/ml by the use of 4 g/l pectinase. After that, a sharp reduction of the activity (46.6 U/ml) was obtained by 4.8 g/l.

### Activation fabric by pectinase

PET/C blended fabrics were initially treated by pectinase enzyme, before loading with ZnO NPs. It has been reported that the biological treatment led to a significant increase of OH and COOH groups on the surface of PET/C blended fabrics^30^. These results were experimentally confirmed by the determination of functional groups existing on the surfaces of PET/C fabrics before and after the activation step. It was found (Table 1) that surface activation leads to an increase in carboxylic content for PET/C blended fabrics, the treatment brings about outstanding increase in carboxylic content from 2.95 to 38.5 meq/100gr to PET/C blended fabrics.

**Table 1:** Effect of the Pectinase Treatment on the Amount of Carboxylic Content of PET/C Blend Fabrics.

Fabrics	Weight Loss %	Carboxylic Content (meq/100 gr. Fabric)
PET/C (Parent)	0.0	3.30
PET/C→ Pectinase	2.95	38.5

Enzymatic Treatment Conditions: [Pectinase]: 5.0 g/l, pH = 5.0, Time, 40 min, Temperature, 45 °C, M: L, 1:15.

**Table 2:** Effect of activation of PET/C blend fabrics on its antimicrobial Activity.

Fabrics	Inhibition zone diameter (mm) in case of loaded Polyester Fabrics* with ZnO NPs				
	Staphylococcus aureus	Pseudomonas aeruginosa	Bacillus subbtilis	Escherichia coli	Candida albicans
PET/C	-ve	-ve	-ve	-ve	-ve
PETC→E	-ve	-ve	-ve	-ve	-ve
PET/C→E→ZnO	50	40	40	35	40

Enzymatic Treatment Conditions: [Pectinase], 5.0 g/l, pH = 4.5, Time, 40 min, Temperature, 45 °C, M: L, 1:15. Treatment Conditions: [ZnO NPs], 5.0%; Curing Temperature, 150˚C; Curing Time, 15 min.* After 5 washing cycles According to AATCC Test Method (61-1989).

-ve = No effect.

**Table 3:** Suppression of pathogens growth by nano particles loaded textile*.

Textile	CFU/ml	
	Candida albicans	Pseudomonas aeruginosa
PET/C (Blank)	1.44 × 10^8^	3.0 × 10^8^
PET/C activated by Pectinase	1.88 × 10^8^	2.2 × 10^8^
PET/C activated and loaded with ZnO NPs	0.84 × 10^8^	0.6 × 10^8^

Enzymatic Treatment Conditions: [Pectinase], 5.0 g/l, pH = 4.5, Time, 40 min, Temperature, 45 °C, M: L, 1:15. Treatment Conditions: [ZnO NPs], 5.0%; Curing Temperature, 150˚C; Curing Time, 15 min.*After 5 washing cycles According to AATCC Test Method (61-1989).

### Loading activated fabric with NPs

**Fig. 6:**
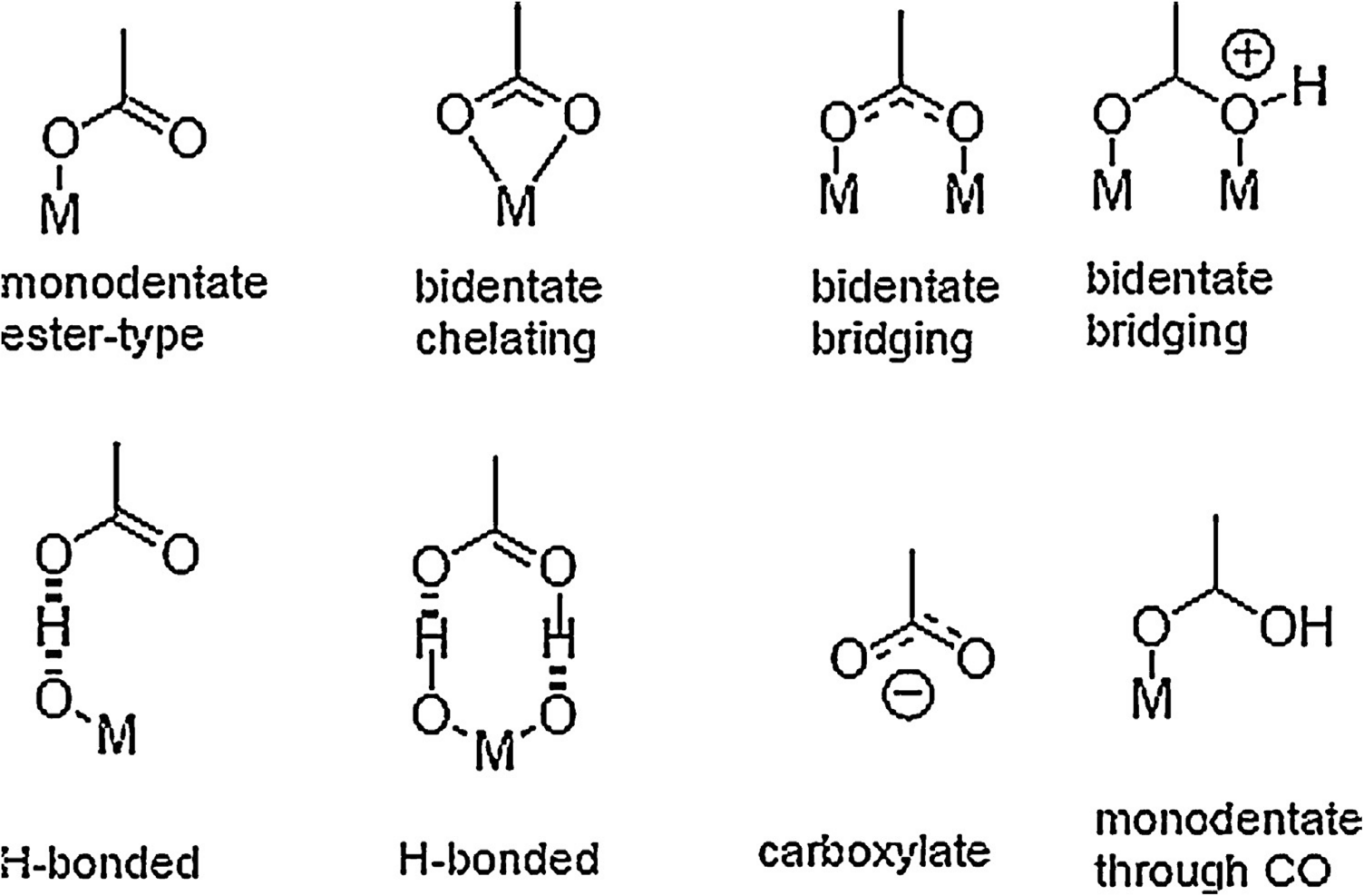
Possible binding mode of a COOH group to NPs [36-37].

The most of the studies relay on the known fact that carboxylic group is the best anchoring group for metals and this is the reason why it was insisted on introduction of new carboxyl groups to different fibers. It is well known that metal (M) atoms can be bound to carboxyl groups through different modes which are shown in Fig.6^41,42^. Carboxyl groups can be bound in a mono-dentate mode to form an ester like linkage or they can bind with each of their two oxygen atoms either to one metal atom (bi-dentate chelating) or to two of them (bi-dentate bridging) (Fig. 6). In addition, they can interact with the metal surface through hydrogen bonding either with a surface bound hydroxyl group and/or a lattice oxygen atom^42^.

### Characterization of PET fabrics loaded with ZnO NPs

#### SEM

**Fig. 7:**
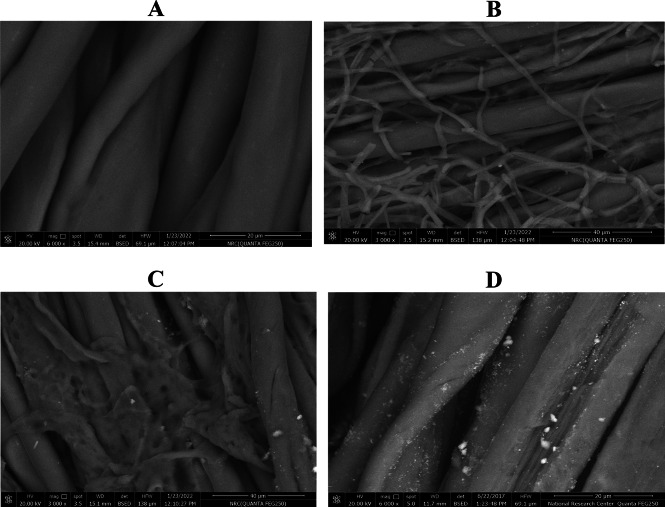
SEM and EDX images of [**A**; **B**; **C** and** D**] Activated and modified PET/C fibrous waste with ZnO NPs (3000X). [**A**] PET/C blended fabric (Parent); [**B**] and [**C**] PET/C blended fabric activated with pectinase (Control); [**D**] PET/C blended fabric activated by pectinase and loaded with ZnO NPs (Treated). After 5 washing cycles According to AATCC Test Method (61-1989).

 The surface topography of PET/C blended fabrics was investigated using SEM technique. Figure 7 shows that the surface of parent PET/C blended fabric was clean and smooth (Fig. 7 A). The treatment of the fabrics with pectinase leads to the formation of some precipitation and random fibers on their surfaces (Fig. 7B, C). Pectinase treatment of PET/C blended fabrics followed by loading with ZnO NPs leads to the formation of a thick layer on the surface of fabrics in the form of coating (Fig. 7D). These findings are in full agreement with the data obtained by EDX technique. The above mentioned changes which took place on the surface topography of PET/C blended fabrics loaded with ZnO NPs are a direct indication that ZnO nanoparticles are attached to the fabric surfaces.

#### EDX

**Fig. 8:**
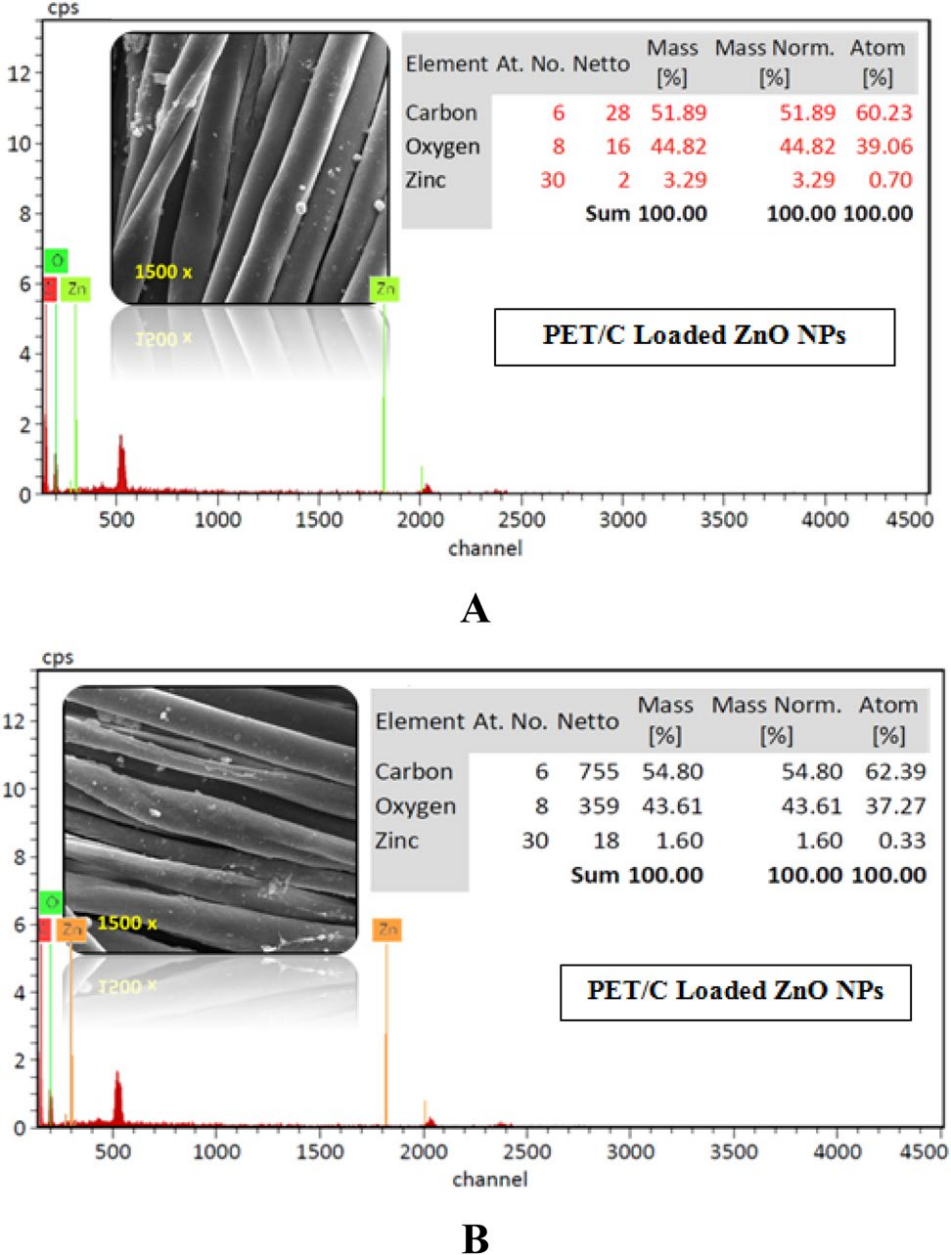
SEM and EDX images of Activated and Loaded PET/C fibrous waste with ZnO NPs (3000X). [A] PET/C blended fabric activated by pectinase and loaded with ZnO NPs (Treated and washed 1 cycle*) [B] PET/C blended fabric activated by pectinase and loaded with ZnO NPs (Treated and washed 5 cycles*) *According to AATCC Test Method (61-1989).

 The presence of ZnO NPs on the surface of PET/C blended fabrics was confirmed by Emission Dispersion X-ray (EDX) analysis. EDX spectra of the PET/C blended fabrics loaded with ZnO NPs following 5 washing cycles are shown in Fig. 8. On the basis of these spectra, it is noteworthy to conclude that the deposited material consisted of Zn and oxygen (Spectra 8 A and 8B). EDX investigation (Fig. 8) showed that even after one and five washing cycles (25 home washings), ZnO is still present on the PET/C blended fabrics surface. Spectra 8 also reveals higher atomic % content on the surface of activated PET/C blended fabrics 3.29 and 1.6 within the two treated and washed samples (Fig. 7A and B). This means that ZnO NPs have sufficient adhesion towards the activated PET/C fabrics under the effect of biological treatment.

It would be of interest to find out whether the loading of ZnO NPs on activated fabrics is through physical or chemical interactions. Therefore, characterization of the so finished PET/C blended fabrics was carried out through scanning electron microscope (SEM) and FTIR.

#### FT-IR

**Fig. 9:**
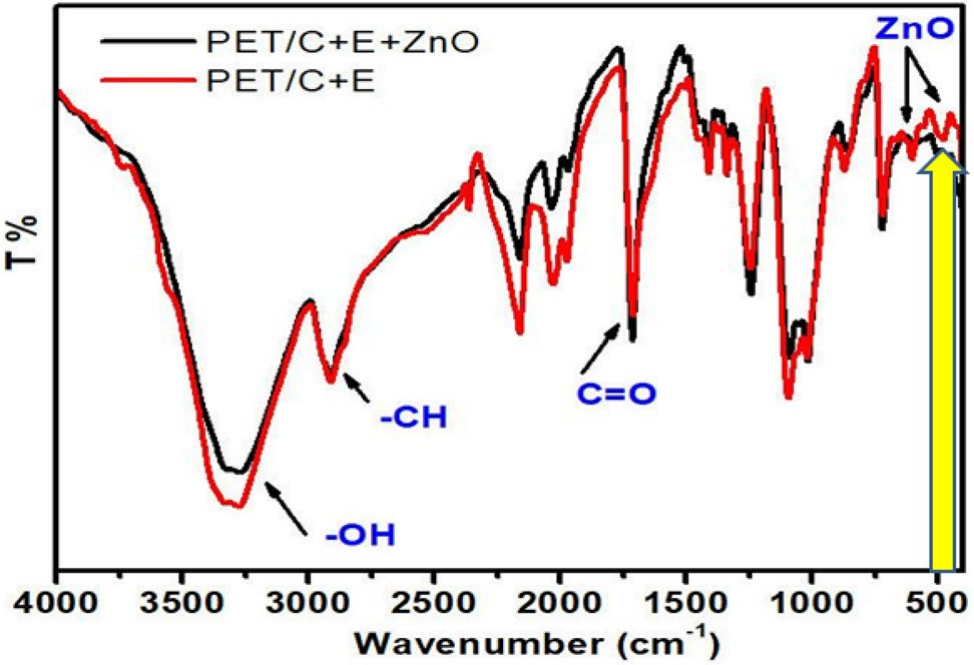
FTIR curves of PET/C blended fabrics activated by pectinase enzyme and loaded with ZnO NPs.

FTIR spectra of parent, activated by pectinase, and finally loaded by ZnO NPs PET/C blended fabrics are shown in Fig. 9. The spectrum of parent PET/C fabrics (Fig. 8) absorptions at 1734, 3432 and 2966 cm^−1^ which are typical to those of > C = O, OH and CH stretching, respectively.

In the spectra of PET/C fabrics activated by pectinase (Fig. 8) we noticed among others the following characteristics signals in the region from 1600 to 1500 cm^−1^ for carboxylate anions (—COO¯). It can be seen that treatment of PET/C blended fabrics with pectinase do not cause any remarkable change in the spectrum (Fig. 8) as compared to parent PET/C blended fabrics except for changes in the relative intensities of the functional groups OH and COOH which indicate that, the active groups have been introduced onto PET/C fabrics surfaces. This finding was confirmed before by carboxylic content measurement, similar finding was reported in^44^.

New absorption peaks at 465 cm^−1^, were observed in the spectrum of PET/C fabrics loaded by ZnO NPs (Fig. 8), which can be due to the attachment of carboxylate anion groups and stretching mode of ZnO NPs. In addition to these results, the characteristics peaks of hydroxyl groups are shifted to lower wave number. The wide peaks at 3427 cm^−1^, corresponding to streaking (—OH) vibration of carboxyl groups moved noticeably to lower wave number at 3419 cm^−1^, and become broader and stronger. This indicates the strong interaction between these groups and ZnO NPs, compared with (Fig. 8), a point which could be explained in terms of strong attachment of ZnO NPs to the COOH groups of activated PET and PET/C fabrics ^43^.

#### Antimicrobial activity

##### Inhibition zone

**Fig. 10:**
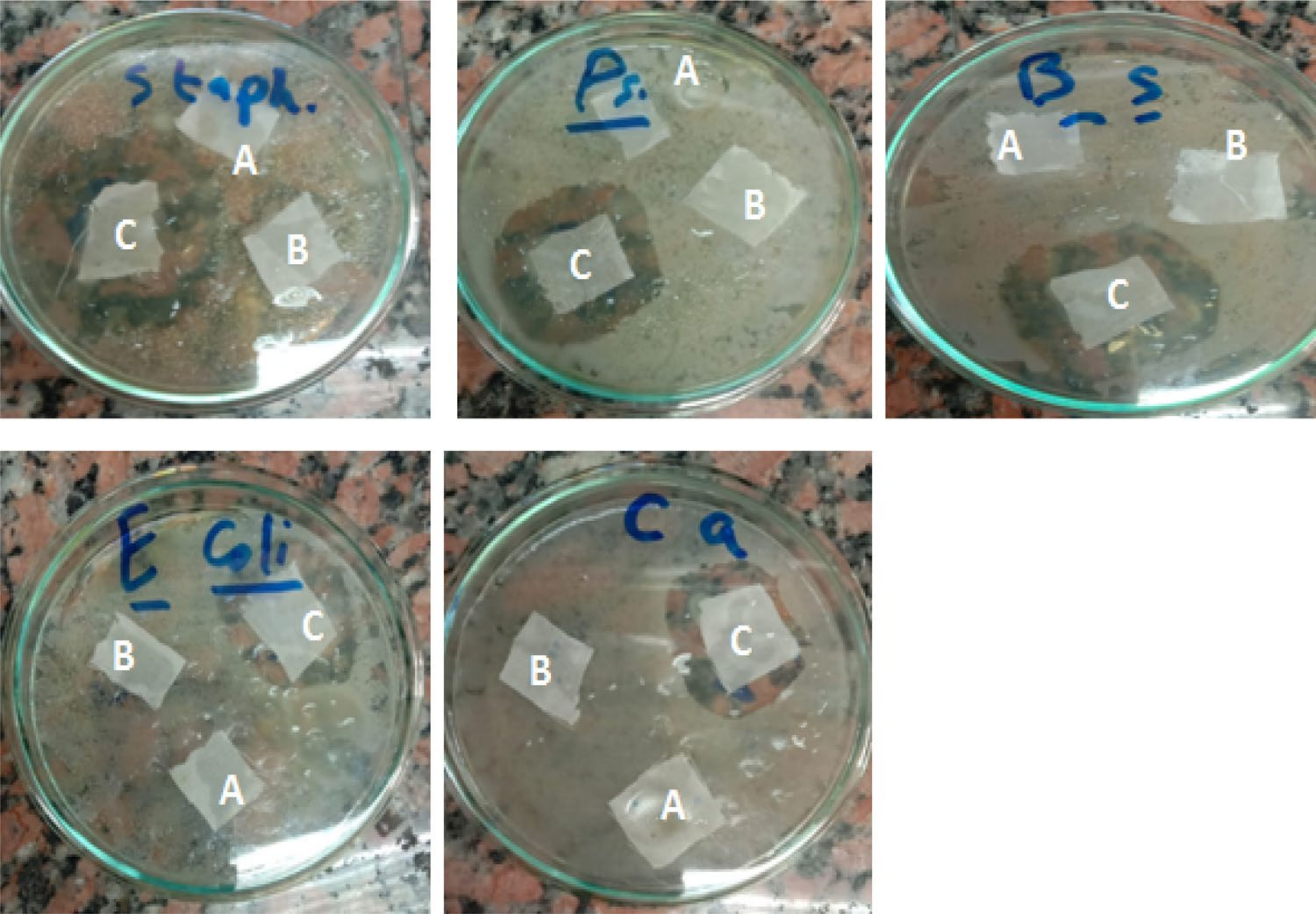
Digital images of inhibition zone of growth over agar plate of PET/C blended fabrics activated by pectinase and loaded with ZnO NPs. [A] PET/C blended fabric (Parent); [B] PET/C blended fabric activated by pectinase (Control); [C] PET/C blended fabric activated by pectinase and loaded with ZnO NPs (Treated*).*After 5 washing cycles According to AATCC Test Method (61-1989).

The antimicrobial activity of PET/C blend fabrics activated with pectinase and loaded with ZnO NPs, was investigated against Gram-positive *Staphylococcus aureus*, *Pseudomonas aeruginosa*, *Bacillus subtilis*, Gram-negative, *E. coli* and non-filamentous fungus *C. albicans*. The activity by diffusion is quantified by the measurement in millimeters of the width of the zone of inhibition around the sample. Table (2) indicates the antimicrobial activity of PET/C blended fabrics loaded with ZnO NPs after activation with pectinase. It is seen from the digital images showed in Fig. 10 that, all PET/C blended fabrics activated and loaded with ZnO NPs after 5 washing cycles, high antimicrobial activity against the previously mentioned microorganisms. In fact, the inhibition zones for all tested modified fabrics samples are significant, whereas no dedication is found for all untreated fabrics. The role of activation of PET/C fabrics with pectinase before loading with ZnO NPs on the antimicrobial activity seems to be more significant as the samples were laundered repeatedly in launder-Ometer^44,45^. This proves the feasibility of the enzymatic activation of PET/C blend fabric on its antimicrobial finishing with ZnO NPs.

##### Evaluation of growth Inhibition of pathogens by cfu/ml (Shake flask Method)

One of the most important industrial advantages of the textile is to assure protection against pathogens that may attack the cloth. When added pieces of fabrics in the fermentation media of the pathogen. CFU/ml was investigated. It was noticed from Table (3) that ZnO Nano particles loaded PET/C blended fabric was superior in protecting the textile material from *Candida albicans* and *Pseudomonas aeruginosa* where 0.84 × 10^8^ and 0.6 × 10^8^ CFU/ml were got respectively, while the textile and the enzyme loaded textile lacked the protection activity.

Zinc oxide NPs disintegrate the microbial cell wall and interact with bio-molecules, leading to the death of the microbe^24^. ZnO NPs may cause cell death by destroying lipids and proteins in bacterial cell membranes, by production of reactive oxygen species (ROS), which are key processes resulting in antibacterial activity. The formation of hydrogen peroxide from ZnO NPs surfaces also leads to inhibition of bacterial growth.

Several methods have been proposed to explain metal ions’ antibacterial action. Metals’ harmful effects on microorganisms have been documented to include antioxidant depletion, DNA damage, poor membrane function, and/or problems with nutrient digestion. Some metal ions, such as Cu^2+^, Co^2+^, Zn^2+^, and Mn^2+^, increase intracellular reactive oxygen species, causing DNA damage, membrane activity disturbance, and suppression of specific enzyme activities required for cell growth. Metal ions bind with the proteins.

Enzymes and other negatively charged groups found in the microorganism’s cell wall and cell membrane cause structural changes that compromise permeability, resulting in cell lysis. Metal ions can enter the bacterial cell and combine with apoenzymes or displace any metal ion required for enzymatic function, limiting protein synthesis and/or hindering nucleic acid synthesis. Metal-catalyzed oxidation of certain amino acids results in a decrease of enzyme catalytic activity. Furthermore, some metals stimulate cell autolytic enzymes, resulting in bacterial death (Zn^2+)^^46^.

#### Ultraviolet protection properties

The effect of activation of PET/C blended fabrics either with pectinase, before loading with ZnO NPs, on UV protection efficiency was investigated. The rate of UV protection was quantified and expressed via UPF values that are given in Table 4. It was found that, the UPF factors for blank PET/C blended fabrics are equal to 10.0. Activation with pectinase followed by the ZnO NPs deposition onto the above mentioned PET/C blended fabrics led to a significant increase in UPF factor to the level corresponding to UPF rating of 50+, which assigns the excellent UV protection. After five washing cycles the UPF values for PET/C blended fabrics were still 50+, which assigns excellent and excellent UV protection. The efficacy of any fictionalization is assessed by its ability to retain the functionality after various washing cycles. To evaluate the washing durability of ZnO NPs functionalized PET/C blended fabrics, washing test was carried out following the AATCC test method 61-1989. The washed fabrics displayed UPF values of + 50 after 5 washes (equivalent to 25 home loundery meter washing cycle), which are still exceptionally high (Table 4). These results show excellent laundering durability of PET/C activated with pectinase and loaded with ZnO NPs.

**Table 4:** Effect of activation of PET/C blend fabrics on its UPF Values.

Fabrics	UPF Values After No of Washing Cycles:			
	1*		5*	
	UPF Value	UPF** Rating	UPF Value	UPF** Rating
PET/C	10.0	Poor	-	-
PETC→ Pectinase	11.1	Poor	11.1	-
PET/C→ Pectinase→ ZnO NPs	54.6	Excellent	51.1	Excellent

Enzymatic Treatment Conditions: [Pectinase], 5.0 g/l, pH = 4.5, Time, 40 min, Temperature, 45 °C, M: L, 1:15. Treatment Conditions: [ZnO NPs], 5.0%; Curing Temperature, 150˚C; Curing Time, 15 min.*According to AATCC Test Method (61-1989). **According to Australia (AS)/New Zealand (NAS) Standard No. 4399 (1996).

## Conclusion

The present study illustrates a facile and environmental friendly method for improving the binding ability of ZnO NPs to PET/C blended fabrics. This method is based on activation the fabrics with biological method by pectinase enzyme before loading fabrics with ZnO NPs by applying pad-dry-cure technique. These loaded fabrics were characterized by SEM, EDX and FT-IR spectroscopy which proved that ZnO NPs was chemically bonded to PET/C blended fabrics. The functionality of PET/C activated and loaded NPs on antimicrobial activity and UV protection efficiency of fabrics was evaluated. The impact of different concentrations of the enzyme and different periods of pectinase incubation with the textile showed the highest activity of the partially purified enzyme (41.4 U/ml) was got at the first 15 min and an increase in the activity of pectin degrading enzyme from 20.5 U/ml by 0.8 g/l enzyme to 70.6 U/ml by the use of 4 g/l pectinase. *Candida albicans* and *Pseudomonas aeruginosa* were got 0.84 × 10^8^ and 0.6 × 10^8^ CFU/ml using shake flask method respectively. Antibacterial activity of finished fabrics against *Staphylococcus aureus*, *Escherichia coli*, *Bacillus subbtilis*, *Pseudomonas aeruginosa* and *Candida albicans* by using inhibition zone revealed superior inhibition zones and excellent UPF rating. The excellent UPF rating, with very less reduction in antibacterial efficiency after 5 washes, indicates the superior washing durability of the finished fabrics with NPs.

## Data Availability

The authors declare that, the data supporting the findings of this study are available within the paper. Should any raw data files be needed in another format they are available from the corresponding author upon request.

## References

[1] Al-Balakocy, N. G., Hassan, T. M., Aly, S. Y., Abd Elsalam, S. H. & Elshakankery, M. H. Using nano technology for imparting PET/C blended fabric new functional performance properties. *J. Eng. Fibers Fabr.* **17**, 15589250221101385. 10.1177/15589250221101385 (2022).

[2] Fiseha, K., Das, D. & Palaniswamy, N. K. Effect of blend ratio of deep-grooved polyester/cotton fibers on mechanical and comfort properties of woven fabrics. *Fibers Polym.* **20**, 2215–2221. 10.1007/s12221-019-9124-4 (2019).

[3] Al-Balakocy, N. G., Hassan, T., Khalil, S. & Abd El-Salam, S. Simultaneous chemical modification and functional finishing of polyester textiles. *Res. J. Text. Appar.* **25** (3), 257–273. 10.1108/RJTA-09-2020-0105 (2021).

[4] Ibrahim, N. A., Eid, B. M., Youssef, M. A., Ameen, H. A. & Salah, A. M. Surface modification and smart functionalization of polyester-containing fabrics. *J. Ind. Text.* **42** (4), 353–375. 10.1177/1528083712440899 (2013).

[5] Radetić, M. & Marković, D. A review on the role of plasma technology in the nano-finishing of textile materials with metal and metal oxide nanoparticles. *Plasma Processes Polym.* **19** (4), 2100197. 10.1002/ppap.202100197 (2022).

[6] Rivero, P. J., Urrutia, A., Goicoechea, J. & Arregui, F. J. Nanomaterials for functional textiles and fibers. *Nanoscale Res. Lett.* **10**, 1–22. 10.1186/s11671-015-1195-6 (2015). 26714863 10.1186/s11671-015-1195-6 PMC4695484

[7] Shah, M. A., Pirzada, B. M., Price, G., Shibiru, A. L. & Qurashi, A. Applications of nanotechnology in smart textile industry: A critical review. *J. Adv. Res.* **38**, 55–75. 10.1016/j.jare.2022.01.008 (2022). 35572402 10.1016/j.jare.2022.01.008 PMC9091772

[8] Periyasamy, A. P., Militky, J., Sachinandham, A. & Duraisamy, G. Nanotechnology in textile finishing: Recent developments. Handbook of Nanomaterials and Nano-composites for Energy and Environmental Applications. :1–31. (2020). 10.1007/978-3-030-11155-7_55-1

[9] Verbič, A., Gorjanc, M. & Simončič, B. Zinc oxide for functional textile coatings: recent advances. *Coatings* **9** (9), 550 (2019). http://www.mdpi.com/journal/coatings

[10] Nisansala, H. M. et al. Zinc oxide nanostructures in the textile industry. *Indian J. Sci. Technol.* **14**, 3370–3395. 10.17485/IJST/v14i46.1052 (2021).

[11] Kathirvelu, S., D’souza, L. & Dhurai, B. A comparative study of multifunctional finishing of cotton and P/C blended fabrics treated with titanium dioxide/zinc oxide nanoparticles. *Indian J. Sci. Technol.* **1** (7), 1–2 (2008). http://www.indjst.org

[12] Kathirvelu, S., D’souza, L. & Dhurai, B. UV protection finishing of textiles using ZnO nanoparticles. *Indian J. Fiber Text. Res. Vol*. **34**, 267–273 (September 2009). https://www.researchgate.net/profile/Kathirvelu-Subramanian/publication/279585204

[13] Palaniappan, G. Study on the antimicrobial efficacy of fabrics finished with nano zinc oxide particles. *J. Text. Cloth. Sci.* **3** (2), 9–15 (2020). https://www.researchgate.net/profile/Gopalakrishnan-Palaniappan-3/publication/370047360

[14] Syduzzaman, M., Hassan, A., Anik, H. R., Akter, M. & Islam, M. R. Nanotechnology for High-Performance textiles: A promising frontier for innovation. *ChemNanoMat* **9** (9), e202300205. 10.1002/cnma.202300205 (2023).

[15] Sfameni, S. et al. Inorganic finishing for textile fabrics: recent advances in wear-resistant, UV protection and antimicrobial treatments. *Inorganics* **11** (1), 19. 10.3390/inorganics11010019 (2023).

[16] Mousa, M. A. & Khairy, M. Synthesis of nano-zinc oxide with different morphologies and its application on fabrics for UV protection and microbe-resistant defense clothing. *Text. Res. J.* **90**, 21–22. 10.1177/0040517520920952 (2020).

[17] Hossain, M. A. & Rahman, M. A review of nano particle usage on textile material against ultra Violet radiation. *J. Text. Sci. Technol.* **1** (3), 93–100. 10.4236/jtst.2015.13010 (2015).

[18] Dejene, B., Kitaw & Tsige Mamo Geletaw A review of plant-mediated synthesis of zinc oxide nanoparticles for self-cleaning textiles. *Res. J. Text. Appar.*10.1108/RJTA-12-2022-0154 (2023).

[19] Ibrahim, N. A. et al. A new approach for durable multifunctional coating of PET fabric. *Appl. Surf. Sci.* **448**, 95–103. https://doi.org/10.1016/j (2018).

[20] Nabil, A., Ibrahim, B. M., Eid, E. A., El-Aziz, T. M. & Abou Elmaaty, S. M. Ramadan loading of chitosan – Nano metal oxide hybrids onto cotton/polyester fabrics to impart permanent and effective multi-functions. *Int. J. Biol. Macromol.* **105**, 769–776. 10.1016/j.ijbiomac.2017.07.099 (2017). 28743573 10.1016/j.ijbiomac.2017.07.099

[21] Radetić, M. & Marković, D. A review on the role of plasma technology in the nanofinishing of textile materials with metal and metal oxide nanoparticles. *Plasma Processes Polym.* **19** (4), 2100197. 10.1002/ppap.202100197 (2022).

[22] Naebe, M., Haque, A. N. M. A. & Aminoddin Haji. and. Plasma-assisted antimicrobial finishing of textiles: A review. *Engineering* 12 : 145–163. (2022). 10.1016/j.eng.2021.01.011

[23] Damuluri, R. & Babel, R. Nanotechnology for antimicrobial textile finishing–A review. Environments. ;7(8). International Journal of Engineering and Techniques - Volume 9 Issue 1, January 2023 (2023). http://www.ijetjournal.org

[24] Al-Balakocy, N. G., Hassan, T., Khalil, S. & Abd El-Salam, S. Simultaneous chemical modification and functional finishing of polyester textiles. *Res. J. Text. Appar.* **25** (3), 257–273. 10.1108/RJTA-09-2020-0105 (2021).

[25] Poortavasoly, H. & Montazer, M. Functional polyester fabric through simultaneous aminolysis and nano ZnO synthesis. *J. Ultrafine Grained Nano-structured Mater.* **47** (2), 113–119 (2014). https://jufgnsm.ut.ac.ir/article_52900_95290572024f3a9d90978528dd1f4685.pdf

[26] Gün Gök, Z., Demiral, A., Bozkaya, O. & Yiğitoğlu, M. In situ synthesis of silver nanoparticles on modified Poly (ethylene terephthalate) fibers by grafting for obtaining versatile antimicrobial materials. *Polym. Bull.* **78**, 7241–7260. 10.1007/s00289-020-03486-9 (2021).

[27] Lv, J., Zhou, Q., Zhi, T., Gao, D. & Wang, C. Environmentally friendly surface modification of polyethylene terephthalate (PET) fabric by low-temperature oxygen plasma and carboxymethyl Chitosan. *J. Clean. Prod.* **118**, 187–196. 10.1016/j.jclepro.2016.01.058 (2016).

[28] Kordoghli, B. et al. UV irradiation-assisted grafting of Poly (ethylene terephthalate) fabrics. *Colloids Surf., A*. **441**, 606–613. 10.1016/j.colsurfa.2013.10.032 (2014).

[29] Ayesh, M., Horrocks, A. R. & Kandola, B. K. The impact of atmospheric plasma/uv laser treatment on the chemical and physical properties of cotton and polyester fabrics. *Fibers* **10** (8), 66. 10.3390/fib10080066 (2022).

[30] Al-Balakocy, N. & Gad Khaled El-Badry, and Talaat Mahmoud Hassan. Multi-finishing of polyester and polyester cotton blend fabrics activated by enzymatic treatment and loaded with zinc oxide nanoparticles. *Cellulose*. London: IntechOpen, 105. (2019). 10.5772/intechopen.89750

[31] Hasan, M. M., Nabi, F. & Mahmud, R. Benefits of enzymatic process in textile wet processing. *Int. J. Fiber Text. Res.* **5** (2), 16–19 (2015).

[32] Kumar, D. et al. Application of enzymes for an eco-friendly approach to textile processing. *Environ. Sci. Pollut. Res.* **14**, 1–1 (2021 Oct). 10.1007/s11356-021-16764-4 34651264

[33] Losonczi, A., Csiszar, E., Szakacs, G. & Kaarela, D. Bleachability and dyeing properties of biopretreated and conventionally scoured cotton fabrics. *Text. Res. J.* **74** (6), 501–508. 10.1177/004051750407400607 (2004).

[34] Rajendran, R., Sundaram, S. K., Radhai, R. & Rajapriya, P. Bioscouring of cotton fabrics using pectinase enzyme its optimization and comparison with conventional scouring process. *Pak J. Biol. Sci.* **14(9**10.3923/pjbs.2011.519.525 (2011). .519 – 25. 10.3923/pjbs.2011.519.525 22032080

[35] Sawada, K., Tokino, S., Ueda, M. & Wang, X. Y. Bioscouring of cotton with pectnase enzyme. *J. Soc. Dyers Colour.* **114** (11), 333–336. 10.1111/j.1478-4408.1998.tb01931.x (1998).

[36] Tzanov, T., Calafell, M., Guebitz, G. M. & Cavaco-Paulo, A. Bio-preparation of cotton fabrics. *Enzym. Microb. Technol.* **29**, 357–362. 10.1016/S0141-0229(01)00388-X (2001).

[37] Yachmenev, V. G., Bertoniere, N. R. & Blanchard, E. J. Effect of sonication on cotton Preparation with alkaline pectinase. *Text. Res. J.* **71** (6), 527–533. 10.1177/004051750107100610 (2001).

[38] Shalaby, S. E., Balakocy, N. G., El-Ola, A. & Beliakova, S. M. Chemical fixation of antimicrobial substance within polyester and Polyamide-6. *Fibers Int. J. Eng. Res. Technol.* **11** (6), 853–865 (2018).

[39] Mojsov, K. Biotechnological applications of pectinases in textile processing and bioscouring of cotton fibers. Inproceedings of II international conference industrial engineering and environmental protection 2012 Oct 31 (pp. 314–322).

[40] Ciechańska, D. & Kazimierczak, J. Enzymatic treatment of fibers from regenerated cellulose. *Fibres Text. East. Eur.* **14** (1(55)), pp92–95 (2006). http://www.fibtex.lodz.pl/55_23_92.pdf

[41] Gambichler, T., Avermaete, A., Bader, A., Altmeyer, P. & Hoffman, K. Ultraviolet protection by summer textiles. Ultraviolet transmission verified by determination of the minimal erythematic dose with Solar-Simulated radiation. *Br. J. Dermatol.* **144**, 484–489. 10.1046/j.1365-2133.2001.04072.x (2001). 11260003 10.1046/j.1365-2133.2001.04072.x

[42] Galoppini, E. Linkers for anchoring sensitizers to semiconductor nanoparticles Coord. *Chem. Rev.* **248**, 1283. 10.1016/j.ccr.2004.03.016 (2004).

[43] Rnsmo, H. et al. *J. Chem. Phys.*, **111** 2744 doi:10.1016/j.ccr.2004.03.016. (1999).

[44] Darwesh, O. M., Matter, I. A. & Al-Balakocy, N. G. MI Abo-Alkasem.Circular economy reinforcement through molecular fabrication of textile wastes with microbial synthesized ZnO nanoparticles to have multifunctional properties. *Sci. Rep.* **14** (1), 16660 10.1038/s41598-024-66430-1 10.1038/s41598-024-66430-1 PMC11271589 39030233

[45] Matter, H. A. G. I. A., Mohamed, I., Abo-Alkasem, N. G., Al-Balakocy, O. M. & Darwesh Phyco-biosynthesis of Chlorella-CuO-NPs and its immobilization on polyester/cotton blended textile waste activated by cellulase enzymes for application as wastewater disinfection filter. *Egypt. J. Chem. Vol*. **67** (7), 609–621. 10.21608/EJCHEM.2024.258806.9102 (2024).

[46] Gad Al-Balakocy, N., El-Ola, A., Mwafy, S. M. & EL-Bendary, E. A. Functional finishing of Polyamide-6 fabrics with Poly quaternary ammonium salt in presence nanometal oxides. *J. Eng. Fibers Fabr.* **15**10.1177/1558925020963000 (2020).

[47] Darwesh, O. M., Al-Balakocy, N. G., Ghanem, A. & Matter, I. A. Application of microalgal-ZnO-NPs for reusing polyester/cotton blended fabric wastes after modification by cellulases enzymes. *Waste Dispos. Sustainable Energy*. **5** (4), 471–482 (2023).10.1007/s42768-023-00170-2

